# Sulfonated Bathocuproine Derivatives as Chemically Bonded Buried‐Interface Engineers for n–i–p Perovskite Solar Cells

**DOI:** 10.1002/advs.76010

**Published:** 2026-06-04

**Authors:** Dong Hyun Kim, Min Ju Jeong, SeungMin Lee, Oui Jin Oh, Mun Young Woo, Sung Yong Kim, Dong Hun Kang, Chan Young Kim, Chan Ho Shin, Jun Hong Noh

**Affiliations:** ^1^ School of Civil Environmental and Architectural Engineering Korea University Seoul Republic of Korea; ^2^ KU‐KIST Green School Graduate School of Energy and Environment Korea University Seoul Republic of Korea; ^3^ Department of Integrative Energy Engineering Korea University Seoul Republic of Korea

**Keywords:** carrier lifetime, chemical bath deposition, molecule, perovskite, sulfonate, Buried Interface, n‐i‐p architecture

## Abstract

Buried‐interface engineering has emerged as a central strategy for advancing the efficiency and stability of halide perovskite solar cells. Bathocuproine (BCP) and its derivatives have been employed as ultrathin insulating interlayers that modulate interfacial junctions and suppress recombination; however, their role has remained limited to passive interface control, and BCP‐based designs have rarely been explored at the electron‐transport‐layer/perovskite buried interface in n–i–p architectures. Here, we elevate the BCP family from a passive junction‐modulating interlayer to a chemically bonded buried‐interface engineer by introducing disodium bathocuproine disulfonate (Na‐BCS) between chemical bath deposited SnO_2_ and FAPbI_3_ perovskite films. Unlike pristine BCP, sulfonate‐functionalized Na‐BCS establishes strong chemical coupling across the buried interface through Na^+^ coordination, sulfonate anchoring to SnO_2_, and pyridinic nitrogen interactions with the perovskite lattice. This molecular bridging simultaneously passivates ETL defects and promotes highly crystalline perovskite growth. Consequently, Na‐BCS incorporation markedly suppresses interfacial recombination, reduces surface recombination velocity, and prolongs carrier lifetime, leading to a 26.02% enhancement in power conversion efficiency and 86% retention of the initial efficiency after 762 h of operation. This work establishes a BCP‐based interfacial design paradigm in which the molecules function as chemically bonded buried‐interface engineers, providing a generalizable strategy for high‐performance n–i–p perovskite solar cells.

## Graphical abstract

A BCP‐derived buried‐interface strategy establishes Na‐BCS as a chemically bonded molecular bridge. By combining sulfonate anchoring to SnO_2_ with preserved BCP–Pb^2^^+^ coordination, Na‐BCS simultaneously passivates oxide‐ and perovskite‐side defects, suppresses non‐radiative recombination, and delivers a surface recombination velocity of 8.6 cm s^−^^1^, a carrier lifetime of ∼2.8 µs, and 26.02% efficiency.

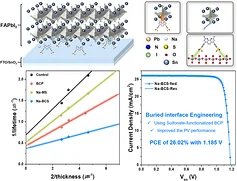

## 1 Introduction

Halide perovskite solar cells (PSCs) have rapidly emerged as a promising next‐generation photovoltaic technology, attaining certified power conversion efficiencies in excess of 26% and continuously demonstrating significant performance enhancements [1, 2, 3]. Interface engineering strategies in PSCs have been demonstrated to effectively passivate trap states, thereby suppressing non‐radiative recombination and modulating the band alignment, ultimately enabling the realization of devices with both high power conversion efficiency (PCE) and superior operational stability [4, 5, 6]. More recently, buried‐interface engineering has been recognized as a particularly critical approach, as it governs the initial formation of the perovskite layer and directly dictates interfacial recombination kinetics [7, 8]. Specifically, buried‐interface engineering at the junction between the electron transport layer (ETL) and the perovskite in n–i–p architectures has been shown to critically influence device performance by enhancing interfacial quality and perovskite crystallinity [9, 10, 11, 12]

In n–i–p architectures, metal oxide such as TiO_2_ and SnO_2_ have been widely employed as ETLs, and notably SnO_2_ has garnered considerable interest owing to its superior hole blocking properties, favorable band alignment, and compatibility with low temperature processing [13, 14, 15]. Among the various SnO_2_ deposition techniques, chemical bath deposition (CBD) has been widely adopted as an ETL, owing to its ability to proceed at temperature below 100 °C and to yield uniform, conformal film growth [13, 16, 17]. Despite these advantages, CBD‐SnO_2_ typically exhibits a high density of surface oxygen vacancies and undercoordinated metal sites, which exacerbate non‐radiative recombination and lead to elevated surface recombination velocity (SRV). Furthermore, unpassivated oxide surfaces can disrupt perovskite nucleation and growth, resulting in compromised crystallinity and shortened bulk carrier lifetimes [18, 19].

Currently, state‐of‐the‐art PSCs have adopted interfacial engineering strategies that combines inorganic salts and molecular modifiers to suppress defect‐mediated recombination and regulate perovskite crystallization [20, 21, 22]. Among molecular interlayers, bathocuproine (BCP) has been extensively used in p–i–n PSCs as an interfacial buffer, most commonly at the top‐side electron‐selective contact [23, 24]. In these configurations, BCP primarily functions as an ultrathin insulating interlayer that modulates interfacial junction properties and suppresses interfacial recombination. In addition, its implementation at buried interfaces has also been explored to improve interfacial quality and device performance. For instance, Fei et al. reported that buried BCP can compete with DMSO for coordination to lead species, thereby reducing residual solvent and suppressing amorphous perovskite formation near the transport interface [25]. In addition, the phenanthroline‐derived nitrogen sites in BCP can interact with undercoordinated Pb‐related species, contributing to reduced interfacial defect density and, in some cases, more favorable crystallization behavior [26, 27]. The limited solubility of BCP in common perovskite precursor solvents further supports its retention at the targeted buried interface during subsequent solution processing [25]. However, despite these advantageous features, BCP and related molecules have been explored predominantly in p–i–n systems, and their rational adaptation to the oxide ETL/perovskite buried interface in n–i–p architectures remains largely unexplored. This is a nontrivial challenge because, unlike in previously reported p–i–n configurations, the n–i–p buried junction requires an interlayer that can simultaneously regulate oxide‐derived interfacial defects and perovskite‐side crystallization.

Herein, we introduce disodium bathocuproine disulfonate (Na‐BCS), a sulfonate‐functionalized derivative of BCP, into the CBD‐SnO_2_/FAPbI_3_ interfacial region to suppress non‐radiative recombination at the buried interface and within the perovskite bulk. In this context, Na‐BCS, a BCP‐derived molecule compatible with the n–i–p architecture, was designed to remain selectively localized at the ETL/perovskite buried interface, where it can simultaneously passivate ETL defects and coordinate with the perovskite through its BCP‐like molecular framework, thereby modulating early‐stage crystallization. Furthermore, to rigorously elucidate the unique attributes of Na‐BCS, BCP, which possesses an analogous chemical framework, and sodium methanesulfonate (Na‐MS), embodying an ionic moiety, were employed for comparative analysis. Unlike pristine BCP, Na‐BCS enables multifunctional chemical coupling across the buried interface. The introduction of Na‐BCS induced chelation between its nitrogen moieties and Pb^2+^, thereby achieving effective passivation and consequently enhancing the crystallinity of the perovskite film. Furthermore, the sulfonate of Na‐BCS was fond to effectively passivate surface oxygen vacancies on the SnO_2_, resulting in a marked decrease in interfacial defect density. Through this chemically bonded molecular bridging, Na‐BCS simultaneously suppresses interfacial and bulk non‐radiative recombination, yielding a low SRV of 8.6 cm s^−^^1^ and an approximately 2.8 µs extension of bulk carrier lifetime. Moreover, pseudo‐JV analysis further corroborated the optimal Na‐BCS condition that minimizes non‐radiative recombination losses. Consequently, the appropriately selected insulating species enabled simultaneous passivation of interfacial and bulk defects, resulting in a power conversion efficiency (PCE) of 26.02%.

## 2 Result and Discussion

**FIGURE 1:**
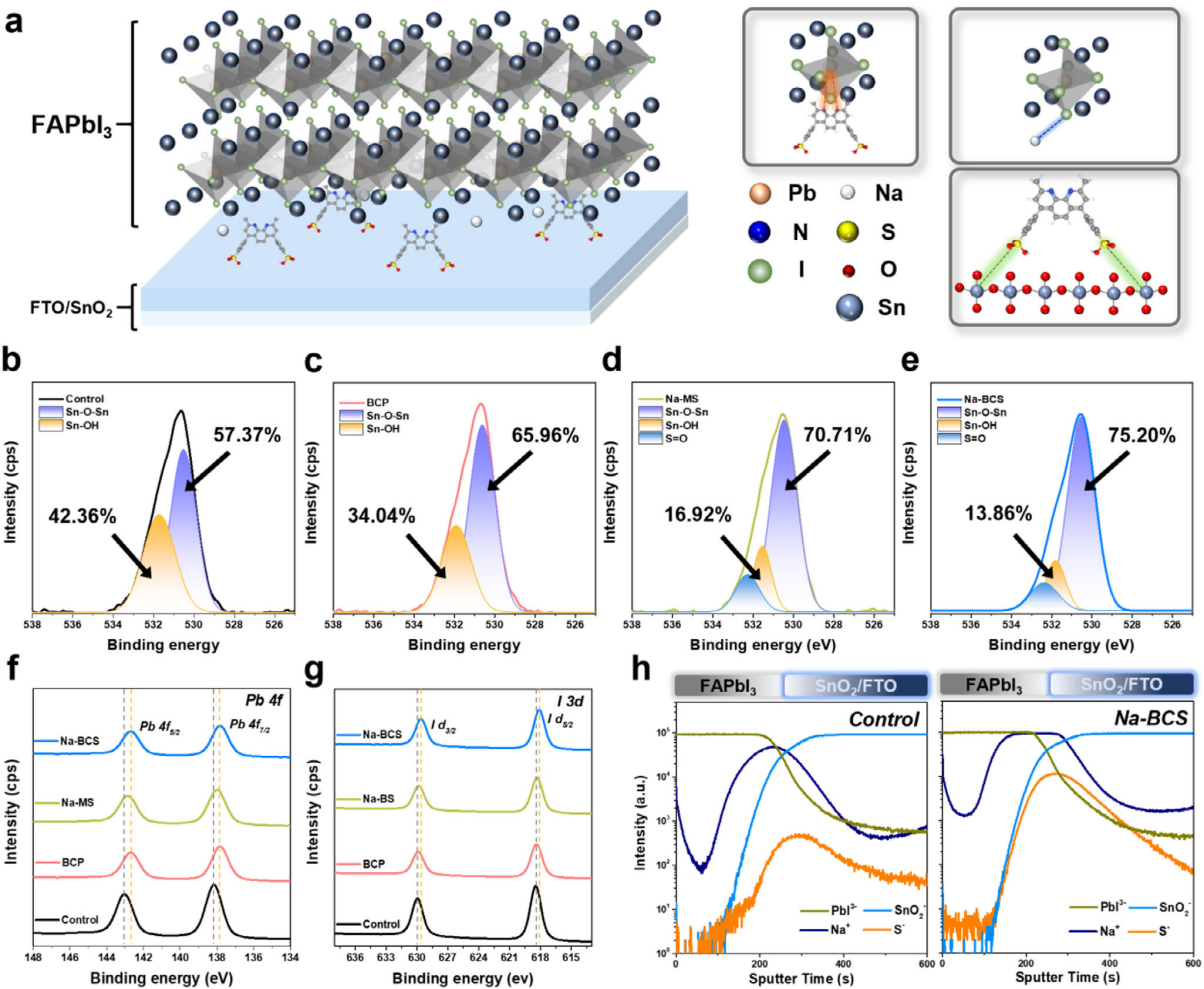
Chemical properties of the buried interface following distinct interface modifications. (a) Schematic illustration of the Na‐BCS mediated passivation mechanism at the buried interface. (b–e) XPS spectra of O 1s for the control and SnO_2_ substrates modified with BCP, Na‐MS, and Na‐BCS. (f and g) XPS of peeled perovskite films for the control, and BCP‐, Na‐MS‐, and Na‐BCS‐modified samples: (f) Pb 4f and (g) I 3d core‐level spectra. (h) TOF‐SIMS elemental depth profiles of the control and Na‐BCS‐incorporated samples at the buried interface.

To effectively suppress defect in both the bulk and interfacial regions of the perovskite layer, Na‐BCS was strategically introduced between the CBD‐SnO_2_ and the FAPbI_3_ perovskite. Furthermore, to rigorously confirm the effectiveness of Na‐BCS treatment, BCP and Na‐MS were introduced under identical processing conditions as reference, thereby enabling a systematic evaluation of their respective roles in interfacial passivation (Figure 1a; Figure S1). Therefore, to gain insight into the interfacial chemical interactions and potential bonding alterations induced by the incorporation of BCP, Na‐MS, and Na‐BCS, X‐ray photoelectron spectroscopy (XPS) was conducted. As shown Figure S2, the Sn 3d_3/2_ and Sn 3d_5/2_ core levels in SnO_2_ films treated with Na‐MS and Na‐BCS exhibited a noticeable shift toward lower binding energies, suggesting alterations in the local chemical environment, while no apparent shift was detected for the BCP‐treated SnO_2_ substrate [28]. The O 1s spectra of the control, BCP‐, Na‐MS‐, and Na‐BCS treated SnO_2_ films were deconvoluted into lattice oxygen (O_L_) and a component corresponding to either oxygen vacancies (O_v_) or surface hydroxyl groups (OH) (Figure 1b–e; Figure S3). Notably, the SnO_2_ films treated with Na‐MS and Na‐BCS exhibited an additional peak associated with the S═O bond of the sulfonate group, which was resolve alongside the O_L_ and (O_v_ + OH) components [29]. These results suggest that, whereas pristine BCP lacks functional groups that can strongly interact with the CBD‐SnO_2_ surface in n–i–p devices, the sulfonate moieties in Na‐BCS enable the intended interfacial anchoring through specific chemical interactions with the ETL. Consistent with this interpretation, the Na‐BCS–treated SnO_2_ exhibited a lower fraction of O_v_‐ and OH‐related species and a higher proportion of O_L_ relative to the control, implying reduced surface recombination and more favorable electron extraction/transport [30]. Additionally, Na^+^ ions were detected exclusively in SnO_2_ films with introduced Na‐MS and Na‐BCS, as identified by XPS analysis (Figure S4). To further probe the chemical interactions induced by the interfacial species on the CBD‐SnO_2_ surface, we carried out complementary FT‐IR measurements (Figure S5). Pristine CBD‐SnO_2_ exhibited a characteristic Sn–O vibrational band at 720 cm^−^^1^ [31, 32]. After introducing BCP, Na‐MS, or Na‐BCS onto the CBD‐SnO_2_ surface, the Sn─O‐related vibration remained observable in all samples. Notably, no discernible shift of the Sn─O band was detected for the BCP‐treated sample. In contrast, Na‐MS‐ and Na‐BCS‐treated films showed distinct sulfonate‐related bands at 1049 and 1045 cm^−^^1^, respectively, together with pronounced shifts of the Sn─O vibration to 711 and 686 cm^−^^1^ [33, 34]. These results are consistent with the XPS observations and indicate a strong sulfonate‐mediated interaction with the SnO_2_ surface. To assess whether this interaction is sufficiently robust to survive post‐treatment, we additionally measured the FT‐IR spectrum after depositing Na‐BCS on CBD‐SnO_2_ followed by a washing process. Importantly, the sulfonate‐related vibrational feature remained clearly detectable even after washing, and the shifted Sn─O band was still observed at 687 cm^−^^1^. The persistence of these features after washing indicates that Na‐BCS is not simply physisorbed on the SnO_2_ surface, but remains strongly anchored through interfacial chemical interaction. Taken together, these FT‐IR results, in conjunction with the XPS analysis, provide further evidence that Na‐BCS forms a robust chemically coupled interface with CBD‐SnO_2_.

Subsequently, FAPbI_3_ perovskite films were deposited on the modified SnO_2_ substrates to form the buried SnO_2_/FAPbI_3_ interface, enabling direct evaluation of chemical interactions on the perovskite side of the junction. XPS analysis was conducted on the buried interface after peeling off the FAPbI_3_ layer from the FTO/SnO_2_/FAPbI_3_ structure. As shown in Figure 1f, the Pb 4_f5/2_ and Pb 4_f7/2_ peaks exhibited a shift toward lower binding energies in the films treated with BCP and Na‐BCS compared to the control film. According to previously reported literature, this result was attributed to the chelation between BCP and Pb^2+^, where the nitrogen in both pyridine groups of the BCP molecule interacted with Pb^2+^ ions [25]. Similarly, the observed shift in the Pb core level peaks in the Na‐BCS‐treated films indicated a comparable coordination interaction. Taken together, these results indicate that sulfonate‐functionalized Na‐BCS not only enables effective anchoring to the underlying ETL but also preserves the intrinsic coordination propensity of the BCP scaffold toward Pb‐related sites in the perovskite, consistent with our multifunctional interfacial design. In addition, Figure 1g shows that the I 3d core level peak in both Na‐MS and Na‐BCS samples exhibited pronounced binding energy shifts, which were attributed to the formation of Na‐I bonds [35]. Based on the previously reported literature, the observed spectroscopic features are consistent with Na‐induced local electronic modulation of the iodide environment at the buried interface, supporting the interpretation that alkali cations can alleviate iodine‐vacancy‐related defects in lead halide perovskites [36, 37]. Given the critical role of iodine‐related defects in defect‐mediated loss and instability in FAPbI_3_, the observed I 3d shift is therefore consistent with Na^+^‐assisted suppression of iodine‐vacancy formation at the buried interface. To further investigate the chemical interaction between FAPbI_3_ and the introduced molecules, we carried out FT‐IR measurements on powder mixtures of FAPbI_3_ with BCP, Na‐MS, and Na‐BCS. Specifically, FAPbI_3_ powder was separately mixed with each molecule in 2‐methoxyethanol, and the solvent was then removed prior to FT‐IR analysis of the resulting powders. As shown in Figure S6a, all mixed powders exhibited the characteristic vibrational features of FAPbI_3_ together with those of the corresponding signals of BCP, Na‐MS, or Na‐BCS, indicating successful mixing of the two components. Notably, the C═N stretching bands of pristine BCP and Na‐BCS were observed at 1621 and 1616 cm^−^^1^, respectively, and shifted to 1606 and 1609 cm^−^^1^ after mixing with FAPbI_3_[25] (Figure S6b). This shift toward lower wavenumber is consistent with electron density redistribution associated with coordination of the pyridinic nitrogen to Pb^2^^+^, thereby supporting a strong interaction between the BCP‐ and Na‐BCS‐containing molecules and Pb^2^^+^ in FAPbI_3_.

The Na‐BCS is localized at the buried interface and interacts specifically with both the ETL and the perovskite layer, we additionally examined the top‐surface XPS spectra of perovskite films in control and BCP‐, Na‐MS‐, and Na‐BCS‐treated samples incorporating these interlayers at the buried interface (Figure S7a). As shown in Figure S7b,c, no Na signal was detected at the top surface of the perovskite layer in any of the samples, and the I 3d core‐level spectra showed no discernible shift across all samples. These results indicate that the Na‐related chemical modulation is localized at the buried interface rather than extending into the perovskite bulk, consistent with an interfacial interaction between Na species and the local iodide environment. Furthermore, whereas the Na‐BCS‐treated sample exhibited a clear Pb 4f core‐level shift at the buried interface, no such shift was observed at the top surface (Figure S7d). Consistent with this, as shown in Figure S7e, no discernible S 2p signal was detected by top‐surface XPS under any condition, indicating that Na‐BCS remains confined to the buried interface rather than diffusing to the perovskite surface. Collectively, these observations demonstrate that Na‐BCS is selectively localized at the bottom interface, where it effectively passivates SnO_2_ defects and promotes interfacial chemical interactions with the adjacent perovskite. To further elucidate the distribution profile of Na‐BCS introduced at the interface, time‐of‐flight secondary ion mass spectrometry (TOF‐SIMS) analyses were conducted on FTO/SnO_2_/FAPbI_3_ samples with and without Na‐BCS (Figure 1h). In Na‐BCS incorporated samples, the concentration of Na^+^ at the buried interface was approximately twofold higher than in control samples, and the Na^+^ ions were localized at the SnO_2_/FAPbI_3_ interface, consistent with previous reports [38, 39]. Additionally, to verify the distribution of the sulfonate of Na‐BCS, the S‐ secondary ion signal was monitored, and it was found to be localized at the SnO_2_/FAPbI_3_ interface without diffusing into the perovskite bulk. The TOF‐SIMS results demonstrates that the incorporation of Na‐BCS at the buried interface effectively preserves the chemical bonding state between the perovskite bulk and the SnO_2_ layer.

**FIGURE 2:**
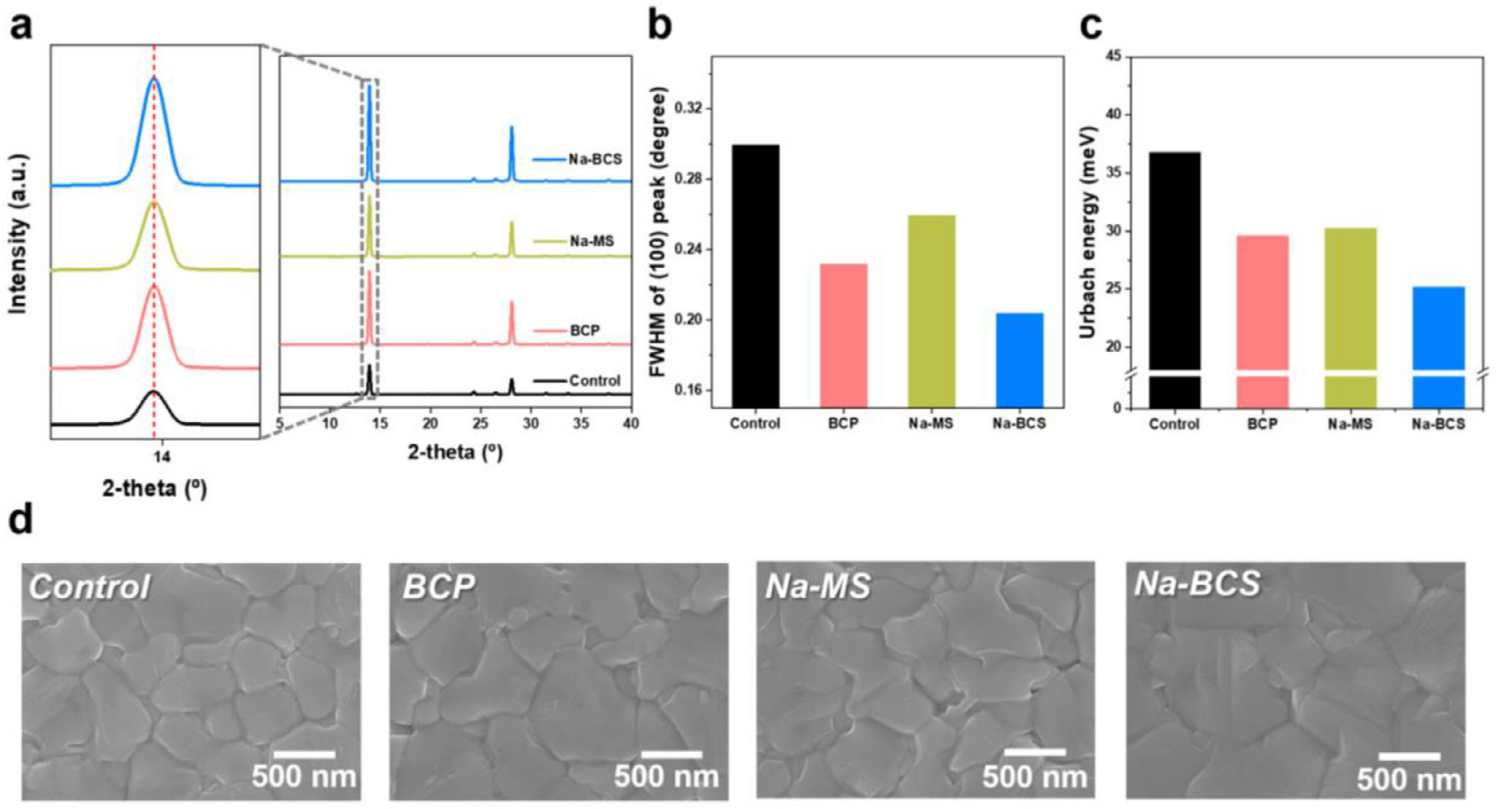
Characterization of perovskite film on BCP‐, Na‐MS‐, and Na‐BCS modified ETL. (a) XRD pattern and (b) FWHM of (100) peak of the FAPbI_3_ films with control, BCP‐, Na‐MS‐, and Na‐BCS incorporated SnO_2_ layer. (c) Urbach energy from UV–vis spectra for control, BCP, Na‐MS, and Na‐BCS samples. (d) Top‐view SEM images for the control, BCP, Na‐MS, and Na‐BCS samples. Scale bar, 500nm.

As illustrated in Figure 2a, to interpret the structural consequences of Pb^2^^+^ chelation by Na‐BCS at the CBD‐SnO_2_/FAPbI_3_ buried interface, we performed XRD analysis on perovskite films prepared with BCP, Na‐MS, and Na‐BCS interlayers. All perovskite films exhibited (100) diffraction peaks consistently positioned near 14°, suggesting that the incorporation of Na^+^ ions from Na‐MS and Na‐BCS had a negligible effect on the lattice parameters. Furthermore, to evaluate the crystallinity of the perovskite films, the full width at half maximum (FWHM) of the (100) diffraction peak was analyzed (Figure 2b). The Na‐BCS‐treated sample exhibited the narrowest FWHM, indicating superior crystallinity and the formation of a high‐quality perovskite film. In addition, to further validate the improvement in perovskite film quality, UV–vis absorption spectroscopy was conducted (Figure S8). The perovskite film incorporating at buried interface exhibited higher absorbance compared to other films, which is consistent with the XRD result, indicating enhanced crystallinity of the film. As shown in Figure S9 and Figure 2C, to gain further into the improved quality of perovskite films, the Urbach energy (E_u_) was calculated from the UV–vis absorption spectra using the relation α = α_0_exp(*hv*/E_u_), where α is the absorption coefficient, α_0_ is a constant, and *hv* represents the photon energy [39]. The perovskite film incorporating Na‐BCS at the buried interface exhibited the lowest Urbach energy among all samples, indicating a lower density of defect states and improved film quality [38]. Furthermore, Tauc plot analysis based on UV–vis absorption spectra revealed that all perovskite films exhibited an identical optical band gap of 1.527 eV, indicating that the incorporation of BCP, Na‐MS, and Na‐BCS did not induce any significant change in the band gap (Figure S10). As shown in Figure 2d, top‐view scanning electron microscope (SEM) images reveal the morphological characteristics of perovskite films deposited on SnO_2_ layers with various interfacial treatments, demonstrating the impact of surface modification on film uniformity and grain structure. All perovskite films showed smooth and compact surface morphologies. Notably, films fabricated with BCP and Na‐BCS treatments exhibited enlarged grain sizes of approximately 2 µm, which is significantly larger than those observed in the control sample, indicating improved crystal growth. Additionally, cross‐sectional SEM analyses were performed on the control sample and on samples incorporating BCP, Na‐MS, and Na‐BCS at the buried interface (Figure S11). In the samples incorporating BCP and Na‐BCS at the buried interface, we observed larger, denser, and more uniform grains in the cross‐sectional SEM images. These results suggest that the interaction at the initial buried interface creates a more favorable condition for perovskite crystal growth, ultimately contributing to improved crystallinity [40].

**FIGURE 3:**
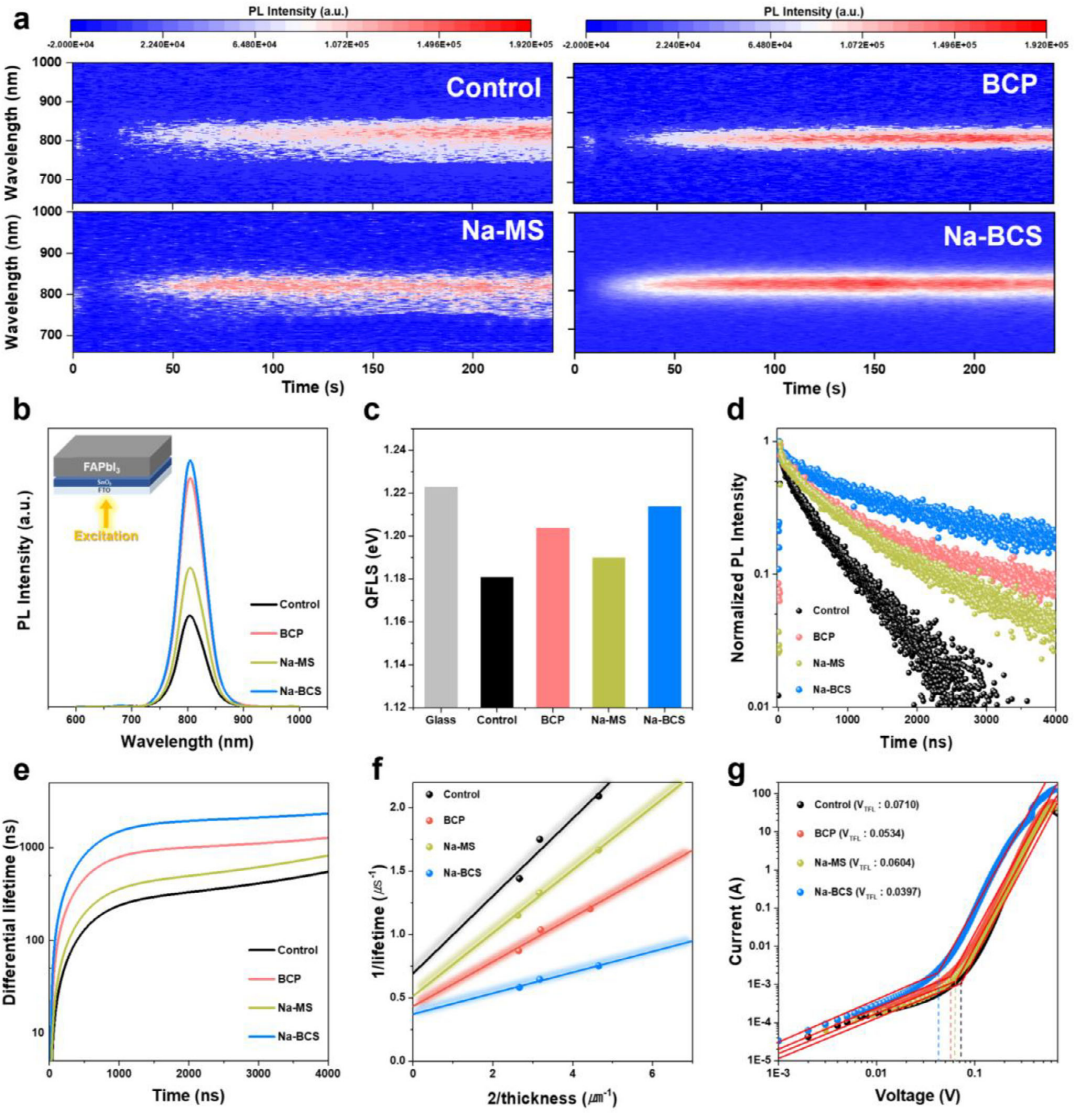
Optoelectronic properties of the buried interface various interfacial conditions. (a) In situ PL mapping of FAPbI_3_ perovskite films with BCP, Na‐MS, and Na‐BCS interlayers, together with the control. (b) Steady‐state PL spectra and (c) calculated QFSL of the control, and BCP‐, Na‐MS‐, and Na‐BCS‐incorporated at buried interface samples. (d) TRPL decay and (e) Differential TR‐PL lifetime for each condition. (f) Bulk lifetime and SRV of the control and BCP‐, Na‐MS‐, and Na‐BCS‐modified buried interface samples as a function of perovskite film thickness. (g) SCLC measurement under dark condition of FTO/SnO_2_/FAPbI_3_/PCBM/Au devices for the control and the interface‐engineered samples.

Furthermore, we performed in‐situ photoluminescence (PL) measurements after introducing BCP, Na‐MS, and Na‐BCS in order to monitor the real‐time crystallization behavior of the perovskite films (Figure 3a). For the control sample, when the photoactive FAPbI_3_ α‐phase emerged after antisolvent treatment, the PL response showed the lowest intensity among all samples together with a relatively broad emission profile. This behavior suggests that the crystallization process proceeded in a less controlled manner, leading to a higher density of defect states associated with non‐radiative recombination and to less uniform crystal growth [41]. The Na‐MS sample exhibited a similarly weak and broadened PL response. By contrast, in the BCP‐ and Na‐BCS‐treated samples, the PL intensity associated with α‐phase formation increased markedly over time. Notably, the Na‐BCS‐treated sample exhibited the highest and most spatially uniform PL intensity during the real‐time crystallization process, together with the narrowest emission linewidth. These observations suggest that Na‐BCS suppresses the formation of non‐radiative defect states more effectively and promotes a more homogeneous crystallization environment [42, 43]. Accordingly, the insitu PL results further support the interpretation that the interfacial interaction between Na‐BCS and Pb^2^^+^ favorably influences the initial nucleation and subsequent crystal‐growth process, thereby contributing to defect‐suppressed, highly crystalline perovskite films [40, 44, 45]. To probe non‐radiative recombination processes at the buried interface in control, BCP‐, Na‐MS‐, and Na‐BCS‐treated films, PL measurements were conducted under excitation from the FTO side (Figure 3b). Among all samples, the introduction of Na‐BCS the interface between CBD‐SnO_2_ and the perovskite layer resulted in the highest PL intensity, suggesting that Na‐BCS effectively passivated interfacial defects and a consequent reduction non‐radiative recombination. To quantitatively evaluate interfacial losses occurring between the SnO_2_ and perovskite, photoluminescence quantum yield (PLQY) measurements were conducted, enabling the subsequent determination of quasi‐Fermi level splitting (QFLS) (Figure 3c). The QFLS of FAPbI_3_ on glass substrate was measured to be 1.223 eV. To evaluate losses at the buried interface, FTO/SnO_2_/FAPbI_3_ were subsequently fabricated. The QFLS values of the control, and the samples with BCP, Na‐MS, and Na‐BCS interfacial modifications were determined to be 1.181, 1.204, 1.190, and 1.214 eV, respectively. Therefore, the Na‐BCS‐modified interface, leveraging the sulfonate‐functionalized BCP scaffold for more effective interfacial binding and passivation, exhibited the lowest V_oc_ loss, consistent with the most efficient suppression of interfacial non‐radiative recombination. To further examine the suppression of non‐radiative recombination through effective interfacial passivation, time‐resolved photoluminescence (TR‐PL) measurements were conducted (Figure 3d; Table S1). The average lifetimes (τ_avg_) of control, BCP‐, Na‐MS‐, and Na‐BCS‐modified samples were measured to be 640.6, 1131.5, 1034.4 and 1812.5 ns, respectively. Notably, the introduced Na‐BCS film exhibited the longest tail lifetime, indicating more effective suppression of non‐radiative recombination at the interface. Additionally, differential TR‐PL were performed to analyze charge extraction and recombination behavior (Figure 3e). The samples modified with BCP, Na‐MS, and Na‐BCS exhibited a steep initial decay in the photoluminescence signal, indicative of rapid and efficient charge extraction at the interface [46]. The sample with introduced Na‐BCS exhibited a distinct lifetime plateau from approximately from 1µs to 4 µs, indicating effective passivation of both the bulk and interfacial regions.

To further elucidate the recombination behavior within the perovskite bulk and at the buried interface, SRV was analyzed by performing TR‐PL measurements as a function of perovskite thickness (Figure 3f; Figure S12). The SRV was calculated based on the double heterostructure model using the following equation:

(1) $$\frac{1}{\tau_{eff}}=\frac{1}{\tau_{b}}+\frac{2SRV}{d}$$

where τ_eff_ is the effective lifetime, τ_b_ is the bulk lifetime, and d represents the thickness of the perovskite layer [47, 48]. The bulk lifetime of the control, BCP‐, Na‐MS‐, and Na‐BCS‐introduced samples were determined to be 1.4, 2.3, 1.9, and 2.8 µs, respectively. The significantly increased bulk lifetime observed in the sample with Na‐BCS indicates an enhancement in perovskite crystal growth and overall bulk quality. As a result, the chelation of the Pb^2+^ by Na‐BCS led to improved crystallinity and overall quality of the perovskite film, as evidenced by the enhanced bulk lifetime. This trend was consistent with previous analyses, including XRD, SEM, and Urbach energy measurements. Furthermore, the Na‐BCS‐introduced sample exhibited the lowest SRV of 8.6 cm s^−1^, reaffirming that the incorporation of Na‐BCS effectively suppressed interfacial defects and mitigated non‐radiative recombination. SRV analysis revealed that Na‐BCS, developed from the BCP framework, concurrently suppressed defect states in the perovskite bulk and at the buried interface, thereby substantiating its dual‐function passivation capability. To examine the passivation effect at the buried interface, space‐charge limited current (SCLC) measurements were conducted using electron‐only devices with the structure FTO/CBD‐SnO_2_/Perovskite/PCBM/Au (Figure 3g). The electron trap density (N_t_) was calculated using the following equation: N_t_ = 2ε_0_εV_TFL_/𝑒𝐿^2^, where ε_0_ is the vacuum permittivity, ε is the relative dielectric constant, V_TFL_ is the trap‐filled limit voltage, e is the elementary charge, and *L* is the thickness of the perovskite layer [49]. The V_TFL_ for the control, BCP‐, Na‐MS‐, and Na‐BCS‐introduced samples were measured to be 0.0710, 0.0534, 0.0604, and 0.0397 V, respectively. Based on these values, the corresponding N_t_ were calculated to be 7.96×10^14^, 5.99×10^14^, 6.77×10^14^, and 4.45×10^14^ cm^−3^, respectively. Taken together, the PL, SRV, and SCLC analyses provide compelling evidence that the introduction of Na‐BCS effectively passivated interfacial defects and enhanced the structural and electronic quality of the perovskite layer.

**FIGURE 4:**
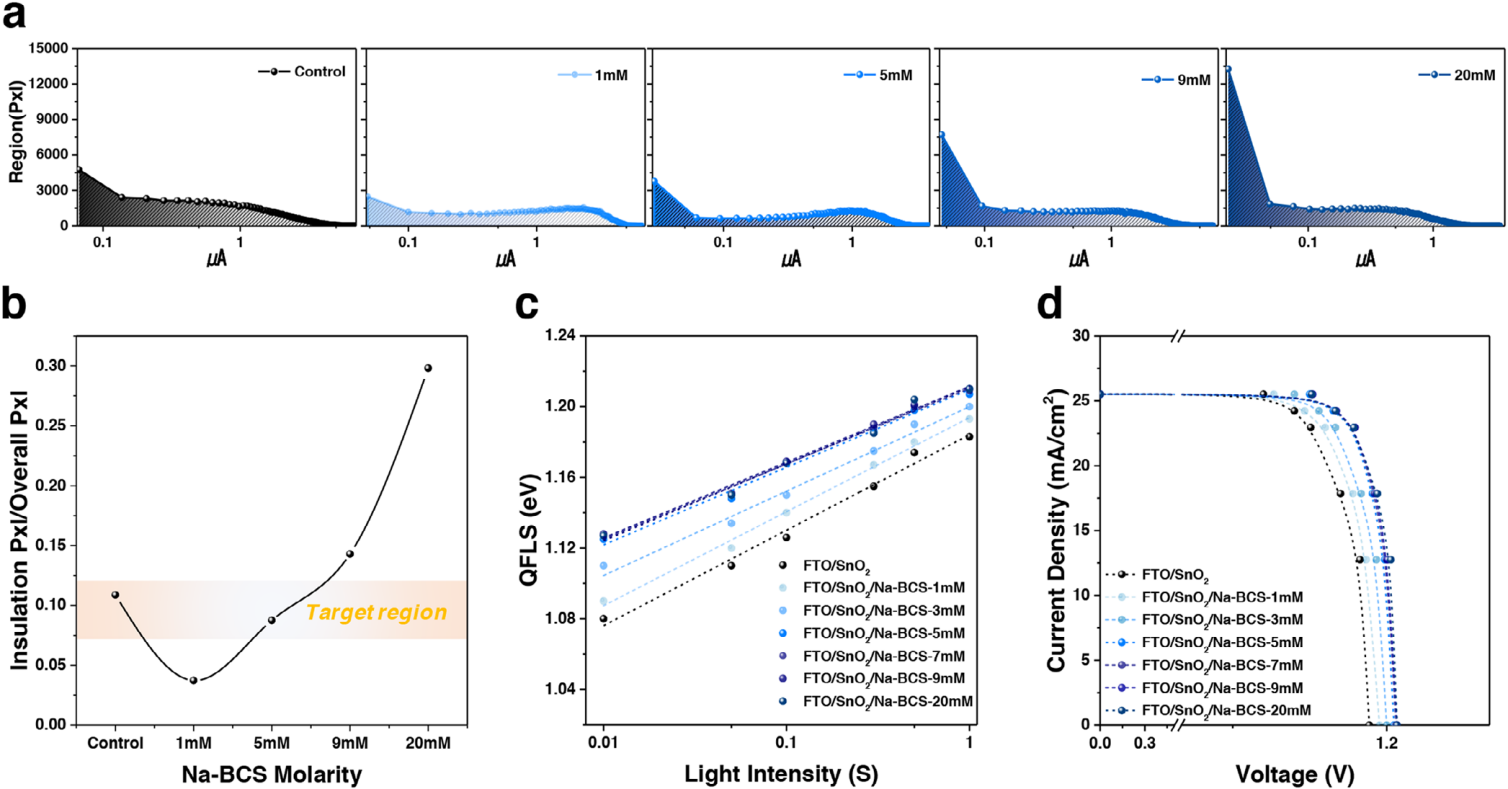
Interface characteristics as a function of Na‐BCS concentration. (a,b) c‐AFM analysis of the effect of Na‐BCS concentration on (a) conductive region pixel and (b) insulation distribution. (c) Intensity dependent QFLS of samples with varying Na‐BCS concentrations, with exponential fitting curves. (d) pseudo‐JV curves derived from intensity dependent QFLS for each condition.

The introduction of Na‐BCS proved well suited to n–i–p architectures, enabling effective simultaneous passivation of both the ETL interface and perovskite bulk. Nevertheless, BCP‐type interlayers are intrinsically insulating, and excessive interlayer thickness or overly strong insulating character can impede interfacial charge transport, ultimately offsetting the passivation benefits. Accordingly, elucidating the trade‐off between transport and passivation for Na‐BCS is important to define processing and design guidelines that maximize its practical benefit in working devices. To this end, we first employed conductive atomic force microscopy (c‐AFM) to quantify the evolution of the local conductivity as a function of Na‐BCS concentration (Figure 4a; Figure S13). Compared with the control, treatment with 1mM Na‐BCS enhanced the electric conductivity of the SnO_2_ layer, which is attributed to sodium ion induced modification of the SnO_2_ lattice [50]. Notably, at Na‐BCS concentrations of 5 mM and above, the fraction of pixels exhibiting currents below 0.1 µA increased sharply, indicating a pronounced increase in the interlayer's insulating character, which is unfavorable for interfacial charge transport. Based on the c‐AFM maps, we further quantified the concentration‐dependent increase in insulating behavior by calculating, over the entire scanned area, the areal fraction of pixels corresponding to the insulating regime (Figure 4b). At Na‐BCS concentrations of 5 mM and above, the area fraction of electronically insulating domains increased relative to the control, suggesting a growing risk of transport limitation at higher loadings. Additionally, We carried out UPS measurements on pristine CBD‐SnO_2_ and on CBD‐SnO_2_ modified with BCP, Na‐MS, and 5 mM Na‐BCS to assess whether the incorporation of 5 mM Na‐BCS introduces any detrimental energetic barrier at the interface (Figure S14). From the secondary electron cutoff analysis, the work functions were determined to be 4.41, 4.40, 4.31, and 4.28 eV for pristine CBD‐SnO_2_, BCP‐, Na‐MS‐, and Na‐BCS‐treated films, respectively. These results indicate that the introduction of Na‐BCS does not induce an unfavorable energetic shift that would be expected to impede electron extraction at the interface. Rather, the modest work‐function shift does not indicate the formation of an additional energetic barrier at the CBD‐SnO_2_/perovskite interface [51]. In light of this trade‐off, to isolate and quantify the passivation benefit of Na‐BCS and identify the optimal treatment condition, absolute PLQY was measured as a function of Na‐BCS concentration under controlled illumination intensities spanning 0.01 to 1 sun. The resulting PLQY values were subsequently converted to QFLS (Figure 4c). Upon exceeding a Na‐BCS concentration of 5 mM, the QFLS plateaued at 1.21 eV, signifying an enhancement of 27.0 meV relative to the control. Furthermore, the light‐intensity‐dependent current density *J*(S) was derived from the excitation intensity measurements, and the corresponding QFLS trends were evaluated (Figure S15). Based on the obtained *J*(S) values, the pseudo‐JV were derived using the relation *pJ*(S) = *J_G_* – *J*(S), where *pJ*(S) denotes the pseudo current density and (S) represents the specific light intensity [52] (Figure 4c). At Na‐BCS concentration above 5mM, the pseudo fill factor (pseudo‐FF) extracted from the pseudo‐JV curves was found to converge to a consistent value of 86.7%. This result suggests that the suppression of non‐radiative recombination becomes saturated at Na‐BCS concentration above 5mM, as pseudo‐JV measurements predominantly reflect recombination losses while excluding transport‐related effects [46]. When considered alongside the C‐AFM measurements, the 5 mM Na‐BCS condition does not markedly strengthen the interfacial insulating character, suggesting that this concentration can mitigate transport‐related losses while maintaining effective passivation.

**FIGURE 5:**
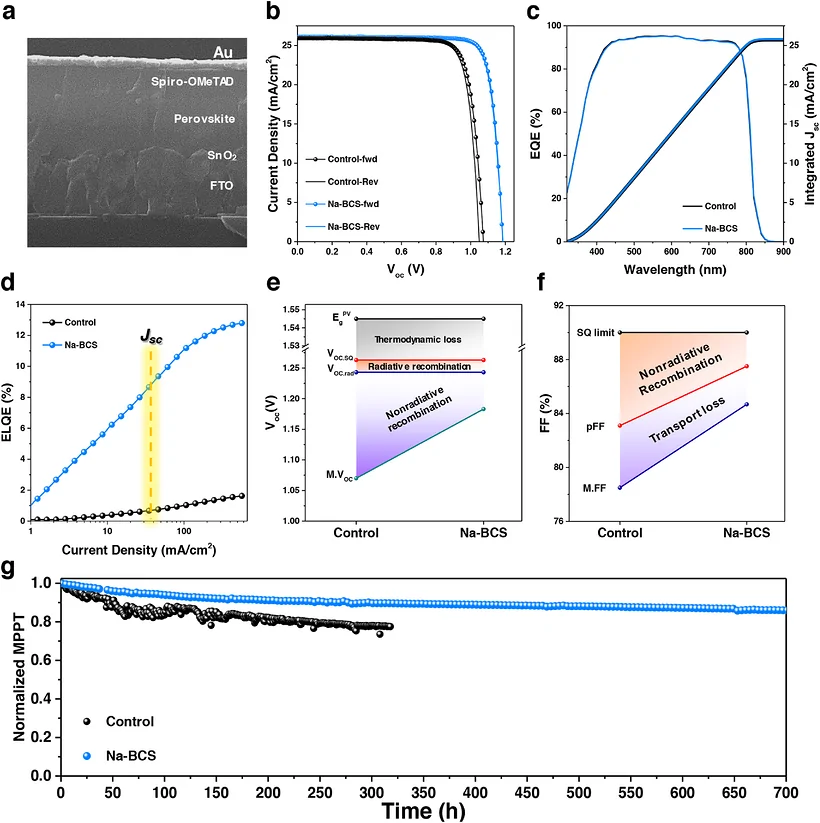
Photovoltaic performance and characterization of PSCs under various conditions. (a) Corrs‐sectional SEM image of representative PSC device. (b) J‐V curves and (c) EQE spectra and integrated *J_sc_* of devices for the control and Na‐BCS, respectively. (d) ELQE of the control and Na‐BCS incorporated devices according to the injected current density. (e) V_oc_ loss analysis and (f) FF loss analysis of the control and Na‐BCS devices. (g) The MPPT of the encapsulated the control and Na‐BCS devices under 1‐sum LED illumination at room temperature and 40% relative humidity, with initial PCE of 22.04% and 25.6%, respectively.

Photovoltaic devices with the architecture of Glass/FTO/CBD‐SnO_2_/FAPbI_3_/OABr/spiro‐OMeTAD/Au were ultimately fabricated with and without the incorporation of Na‐BCS (Figure 5a). As shown in Figure 5b and Table S2, the current density‐voltage (*J*‐*V*) curves for the control and for devices incorporating Na‐BCS at systematically varied concentrations during fabrication. In the forward scan, the control device exhibited a *J_sc_* of 25.93 mA cm^−2^, V_oc_ of 1.073 V, and a FF of 80.62%, resulting in a PCE of 22.43%. At its optimized concentration, the 5mM Na‐BCS device delivered a remarkable PCE of 26.02% under forward scan conditions, with a *J_sc_* of 26.19 mA cm^−2^, a V_oc_ of 1.185 V, and a FF of 83.83%, thereby demonstrating significant improvement in photovoltaic performance (Table 1). Notably, consistent with the C‐AFM and pseudo‐JV analyses, the 5 mM Na‐BCS condition exhibited an optimized passivation effect while minimizing charge‐transport losses. The integrated *J_sc_* from external quantum efficiency (EQE) data was 25.5 and 25.8 mA cm^−2^ for the control and Na‐BCS device, respectively, which is consistent with the *J*‐*V* derived current density (Figure 5C). Devices employing BCP and Na‐MS as interfacial modifications achieved optimized PCE of 25.16% and 25.07%, respectively (Tables S3 and S4). As shown in Figure S16, the distribution of the photovoltaic parameters was analyzed for all samples. Notably, the Na‐BCS‐treated devices consistently delivered the highest PCE among the tested conditions, while also exhibiting a comparatively tighter distribution of device performance. To quantitatively assess the V_oc_ losses in both the control and Na‐BCS devices, electrochemical quantum efficiency (ELQE) analysis was conducted (Figure 5d). At an injection current density near the *J_sc_*, the control and Na‐BCS devices exhibited ELQE values of 0.74 and 9.3%, respectively. These results show consistency with corresponding trends in PLQY, SRV, and pseudo‐JV, collectively reinforcing the interpretation of improved interfacial and radiative properties. The BCP and Na‐MS devices exhibited ELQE values of 6.5 and 1.4%, respectively, aligning closely with the V_oc_ enhancements observed relative to the control device (Figure S17). Furthermore, the V_oc_ was analyzed as a function of the logarithm of the light intensity according to the equation “V_oc_ = n_id_(k_B_T/q)ln(light intensity) + α”, where n_id_, k_B_, T, q, and α represent the ideality factor, Boltzmann constant, temperature, elementary charge, and an constant, respectively (Figure S18). The extracted slope indicated a suppression of non‐radiative recombination pathways. The Na‐BCS device exhibited a reduced slope in the V_oc_ versus ln(light intensity) plot compared to the control, BCP, and Na‐MS devices. This notable reduction in slope indicates that the incorporation of Na‐BCS effectively suppresses non‐radiative recombination within the full device architecture, thereby enhancing charge carrier retention and overall photovoltaic performance. As shown in Figure 5e,f, at the optimized Na‐BCS concentration of 5 mM—where non‐radiative recombination is effectively suppressed without incurring appreciable transport‐related losses—the Na‐BCS device exhibits clear improvements in V_oc_ and FF relative to the control, corroborating enhanced interfacial passivation and optoelectronic quality upon Na‐BCS incorporation.

Finally, to evaluate the photostability of the optimized Na‐BCS device, both the control and Na‐BCS devices were encapsulated and subjected to continuous illumination under ambient conditions (Figure 5g). In contrast to the control device, which exhibited a T_80_ lifetime of 243 h, the Na‐BCS device retained 86% of its initial performance after 762 h of continuous illumination. To investigated the improved photostability imparted by Na‐BCS, encapsulated FTO/CBD‐SnO_2_/FAPbI_3_ samples with and without Na‐BCS were subjected to continuous illumination, and the temporal evolution of their PL was monitored (Figure S19). With prolonged illumination, the control device exhibited a progressive decrease in PL intensity. These results suggest that prolonged illumination induces an increase in interfacial trap density at buried interface, which accelerates non‐radiative recombination and destabilizes the FAPbI_3_ crystal structure. In contrast, the Na‐BCS sample showed a pronounced increase in PL intensity over the first 30 h of illumination, and, unlike the control, exhibited no discernible PL decay even after 100 h of continuous light soaking. These trends suggest that non‐radiative recombination at the buried interface is effectively suppressed under operating conditions. To further examine whether Na‐BCS remains localized at the buried interface under operating conditions, TOF‐SIMS analysis was carried out on devices subjected to 1‐sun MPPT photostability testing. As shown in Figure S20, devices with an FTO/SnO_2_/FAPbI_3_/Spiro‐OMeTAD/Au architecture, with Na‐BCS at the buried interface, were subjected to MPPT tracking under 1‐sun illumination. The Na‐BCS devices aged 275 h were subsequently analyzed by TOF‐SIMS. TOF‐SIMS analysis revealed that the Na^+^ signal exhibited partial redistribution into the perovskite bulk relative to its initial depth profile before operation. By contrast, the S‐related secondary‐ion signal remained strongly concentrated at the SnO_2_/FAPbI_3_ buried interface after device operation. These results suggest that, although Na^+^ undergoes some redistribution during operation, the sulfonate‐functionalized molecular framework remains effectively anchored at the buried interface, consistent with persistent interfacial coupling to the SnO_2_ surface. When considered together with the PL results, this finding supports the interpretation that the buried‐interface passivation effect associated with Na‐BCS is retained under operating conditions, thereby contributing to suppressed non‐radiative recombination and enhanced device photostability.

**TABLE 1:** Photovoltaic parameters of the control and Na‐BCS devices, respectively.

Devices	Scan direction	J_sc_ (mA cm^−2^)	V_oc_ (V)	FF (%)	PCE (%)	R‐F average PCE (%)
Control	Fwd.	25.93	1.073	80.62	22.43	22.44
	Rev.	26.18	1.056	81.20	22.45	
Na‐BCS	Fwd.	26.19	1.185	83.83	26.02	25.94
	Rev.	26.21	1.186	83.25	25.87	

## 3 Conclusion

In this study, incorporating Na‐BCS, a sulfonate‐functionalized BCP derivative, at the buried interface of n–i–p perovskite solar cells simultaneously passivated SnO_2_ surface defects and promoted higher‐quality perovskite crystallization, thereby enabling devices with outstanding photovoltaic performance. Notably, we confirmed that the sulfonate functionalities of Na‐BCS effectively passivate oxygen vacancies in SnO_2_ layer and that its nitrogen sites chelate Pb^2+^ ions within the perovskite, resulting in a pronounced enhancement of perovskite crystallinity. These results demonstrate that introducing sulfonate functionality extends the interfacial scope of the BCP scaffold beyond its conventional role by delivering coordinated crystallization control together with effective SnO_2_ defect passivation. In addition, elucidating the correlation between the insulating character and the passivation efficacy of Na‐BCS enabled the rational optimization of the interlayer, maximizing defect passivation while minimizing transport penalties associated with excessive electrical insulation. This buried‐interface engineering strategy markedly prolonged the bulk carrier lifetime and reduced the surface recombination velocity, delivering devices with an open‐circuit voltage exceeding 1.18 V and a power conversion efficiency of 26.02%. Accordingly, this interface‐specific and chemically anchored Na‐BCS interlayer transforms BCP‐derived modifiers from passive junction‐tuning layers into active buried‐interface engineers that suppress defect‐mediated recombination while preserving efficient charge extraction. By establishing a BCP‐based interfacial design paradigm, this work offers a general framework for deploying chemically anchored BCP derivatives at buried interfaces in high‐efficiency n–i–p perovskite solar cells.

## 4 Materials

Tin(II) chloride dihydrate, urea, hydrochloric acid (HCl, 37%), thioglycolic acid (TGA), N,N‐dimethylformamide (DMF), dimethyl sulfoxide (DMSO), and chlorobenzene (CB) were purchased from Sigma Aldrich. Lead iodide, BCP, and Na‐BCS were purchased from Tokyo Chemical Industry. Formamidinium iodid (FAI), methyl ammonium chloride (MACl), and n‐Octylammonium bromide (OABr) were purchased from Greatcell Solar. 2,2’,7,7’‐tetrakis[N,N‐di(4‐methoxyphenyl)amino]‐9,9’‐spirobifluorene (Spiro‐OMeTAD) was purchased from Lumtec. All the materials were used without further purification.

### 4.1 Device Fabrication

FTO substrates (Asahi glass) were etched using zincpowder and HCl. The etched substrates were cleaned with deionized water, acetone, ethanol, and 2‐propanol, each for 15 min. The substrates were treated UV/O_3_ for 30 min to deposit tin oxide (SnO_2_) through Chemical bath deposition (CBD). Sequentially, 1.75 g of SnCl_2_·2H_2_O and 10 g of urea were dissolved in a mixture of 200 µL of thioglycolic acid, 10 mL of HCl, and 800 mL of deionized water. The FTO substrates were immersed in this solution at 75°C for 12 h. Afterward, the SnO_2_ deposited substrates sere washed with deionized water for 10 min and annealed at 150°C for 1 h. Na‐MS and Na‐BCS were dissolved in D.I depending on different molarity and the different molarity of BCP were dissolved in CB, respectively. The prepared solution spin‐coated on the SnO_2_ substrates at 5,000 rpm for 30 s, followed by annealing at 150°C for about 10 min. 1.7 M FAPbI_3_ perovskite solution was prepared by dissolving FAI (1.7 m), PbI_2_ (1.7 m), MACl (35 mol %) in mixed solvent of DMF: DMSO = 8:1 v/v. The 70 µL of perovskite solution was then spin‐coated onto the substrates at a spin rate of 500 rpm for 3 s, 1000 rpm for 5s, and 5000 rpm for 10s, and 1mL of ethyl ether was dropped on the substrates during the spin‐coating. Subsequently, the substrates were annealed at 120°C for 60 min. 1.6 mg of OABr was dissolved in 1mL of chloroform and spin‐coated onto the perovskite film at 5,000 rpm for 30s. Then, the films were annealed at 100°C for 5 min. The sprio‐OMeTAD solution was prepared by dissolving 0.1 g of spiro‐OMeTAD, 23 µL of Li‐TFSI solution (540 mg mL^−1^ in acetonitrile), 10 µL of tris(2‐(1Hpyrazol‐1‐yl)‐4‐tert‐butylpyridine)cobalt(III) tri[bis(trifluoromethane)sulfonamide] (Co‐TFSI) solution (375 mg mL^−1^ in acetonitrile), and 39 µL of 4‐tertbutylpyridine in 1.1 mL of CB. The prepared solution was spin‐coated on the FTO/SnO_2_/Perovskite substrates at 2,000 rpm for 30 s.

### 4.2 Film Characterization

SEM measurements were carried out FE‐SEM (Regulus8230). The optical properties of the films were measured through UV–vis spectroscopy (Cary 5000, agilent Technologies). XRD spectra were measured by using a Rigaku Dmax2500‐PC with an X‐ray tube (copper K_α_, λ = 1.54 Å, 200 mA, 40kV, 8kW). XPS analysis was measured by using Ulvac PHI (PHI 5000 VersaProbe) equipment with Al Kα as the X‐ray source and a beam diameter of 100×100 µm. TOF‐SIMS meassurement was conducted by using a time‐of‐flight secondary ion mass spectrometer (ION‐TOF GmbH) equipped with a 5 keV Ar^−^ ion beam for the sputtering and 30 keV Bi^+^ plused primary ion beam for the analysis. Conductive‐AFM measurements were carried out by uisng a FX40. The steady‐state PL was measured using a 485 nm diode laser (Horiba, DeltaDiode‐485 L). Time‐correlated single photon counting (TCSPC) was implemented using the pulsed mode of the diode laser with 485 nm (Horiba, DeltaDiode‐485L‐CW). The photon for the film was collected using the double‐grating monochromator (Horiba, FL‐1005) and a liquid‐nitrogen‐cooled low noise phtomultiplier tube (Hamamastsu, R5509‐43). Electron‐only devices with the structure FTO/SnO_2_/FAPbI_3_/PCBM/Au were fabricated under each condition for space‐charge‐limed current (SCLC) measurements. SCLC measurements of the control, BCP, Na‐MS, and Na‐BCS devices were conducted by increasing the bias from ‐5 to 10V at a scan rate of 50mV/s at room temperature under dark condition.

### 4.3 Device Characterization

*J*‐*V* curves were measured using asolar simulator (Newport, oriel Class A, 91195A) with asource meter (Keithley 2420) under illumination at AM 1.5G, which was calibrated using a Si‐reference cell certificated by the National Renewable Energy (NREL). For the *J*‐*V* curve characterization, step voltage and scan speed were fixed at 10 mV and 100 mV s^−1^, respectively. For the measurement of high performance devices, we attached an anti‐reflection film to the back side of the device. The active area was determinded by the metal masks with circle‐type (0.096 cm^2^) placed in front of the devices. The EQE was measured using QUANTX‐300 QE measurement with a 100 W xenon lamp source and a 130 mm focal length monochromator with dual gratings. ELQEs were measured by directly attaching a calibrated silicon photodiode (Hamamatsu, S1227‐1010BQ), which was large collection area than the active area of device. The inject current of the source meter unit (Keithley 2450) and the detected photocurrent of the photodiode were Controlled and measured using the customized program.

## Author contributions

D.H.Kim and J.H.N. conceived the idea and designed the experiments. D.H.Kim, M.J.J., S.M.L. and O.J.O. contributed equally to this work. D.H.Kim and J.H.N wrote the paper. D.H.Kim and M.J.J. fabricated the single‐junction. S.M.L. and O.J.O. conducted PL and TRPL analyzed. D.H.Kang and H.C.S conducted SCLC analyzed. All authors read and commented on the paper.

## Conflicts of Interest

The authors declare no conflict of interest.

## Supporting information




**Supporting File**: advs76010‐sup‐0001‐SuppMat.docx.

## Data Availability

The data that support the findings of this study are available from the corresponding author upon reasonable request.
