# Supplementary material for: Sulfonated Bathocuproine Derivatives as Chemically Bonded Buried‐Interface Engineers for n–i–p Perovskite Solar Cells
Source: Adv Sci (Weinh). 2026 Jun 4:e76010. Online ahead of print. doi: 10.1002/advs.76010 (PMC13336789; doi:10.1002/advs.76010)
Supplement: Supplementary file 1 — Supporting File: advs76010‐sup‐0001‐SuppMat.docx. [file ADVS-9999-e76010-s001.docx]

**Supporting Information**

**Sulfonated Bathocuproine Derivatives as Chemically Bonded Buried-Interface Engineers for n–i–p Perovskite Solar Cells**

Dong Hyun Kim^1†^, Min Ju Jeong^1†^, Seung Min Lee^1†^, Oui Jin Oh^1†^, Mun Young Woo^1^, Sung Yong Kim^1^, Dong Hun Kang^1^, Chan Young Kim^1^, Chan ho Shin^1^ and Jun Hong Noh*^1,2,3^

^1^ School of Civil, Environmental and Architectural Engineering, Korea University, Seoul. 02841, Republic of Korea

^2^ KU-KIST Green School Graduate School of Energy and Environment, Korea University, Seoul 02841, Republic of Korea

^3^ Department of Integrative Energy Engineering, Korea University, Seoul 02841, Republic of Korea

*Corresponding author: Email: [junhnoh@korea.ac.kr](mailto:junhnoh@korea.ac.kr) (J. H. N.)


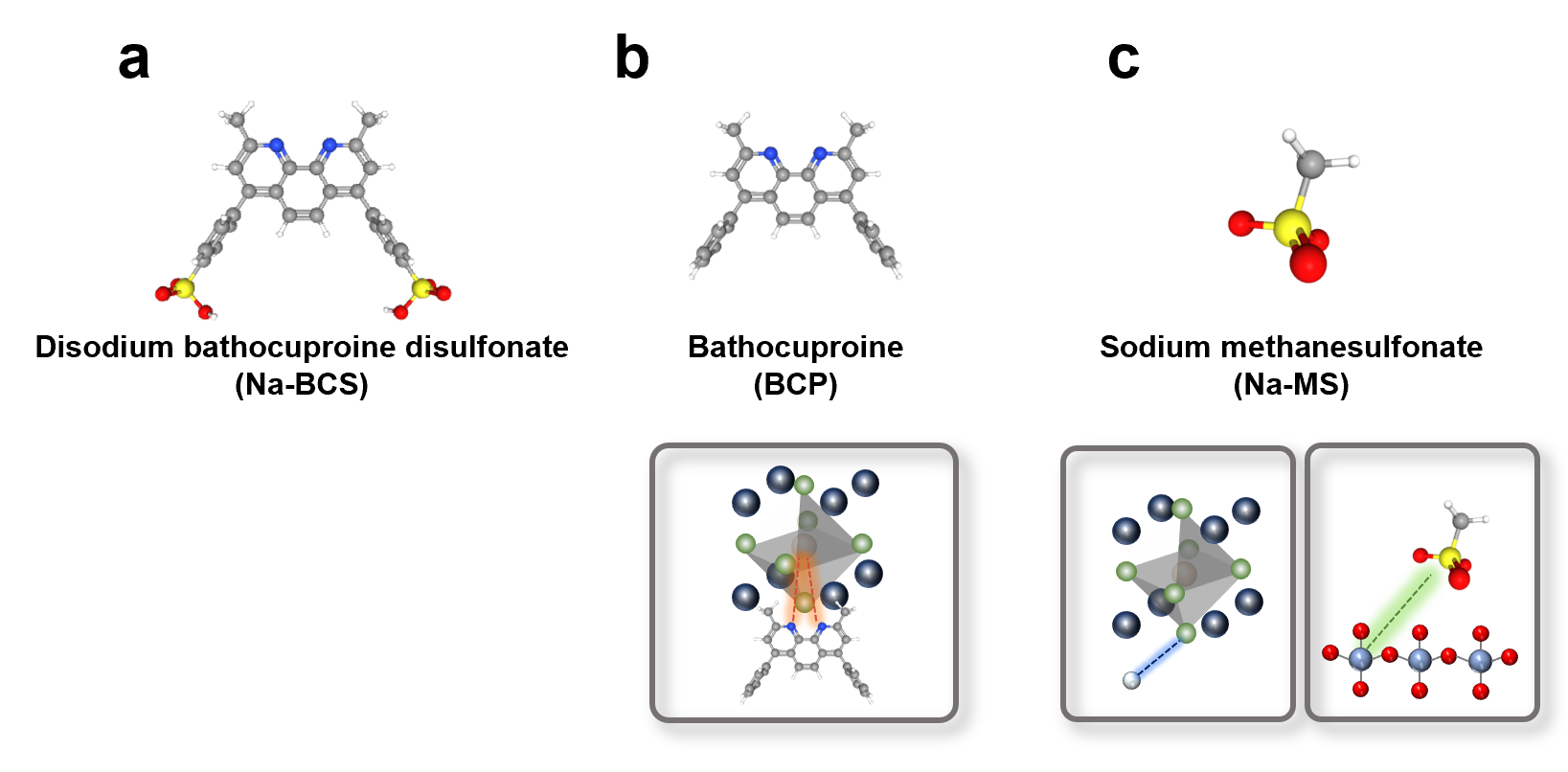
**Figure S1**. Schematic illustration of n–i–p perovskite solar cell configuration and structure of incorporated materials on ETL.

.

**Figure S2**. XPS of the Sn 3d core levels for the control and SnO_2_ substrates modified with BCP, Na-MS, and Na-BCS

**Figure S3**. Relative ratio of deconvoluted XPS O 1s peak components for each sample.

**Figure S4**. XPS of the control, and BCP-, Na-MS-, and Na-BCS-modified SnO_2_ for detection of Na 1S.

*
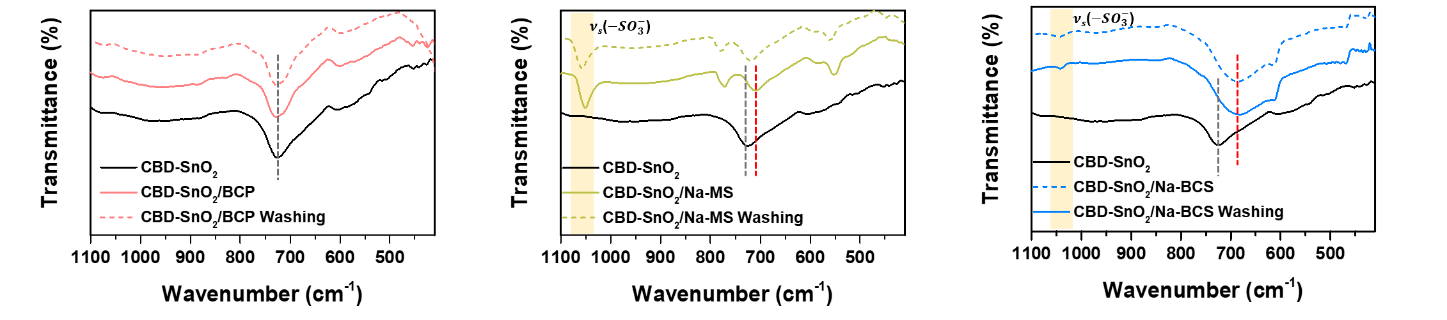
***Figure S5**. FT-IR spectra of pristine CBD-SnO₂, CBD-SnO₂ treated with BCP, Na-MS, or Na-BCS, and the corresponding films after washing.


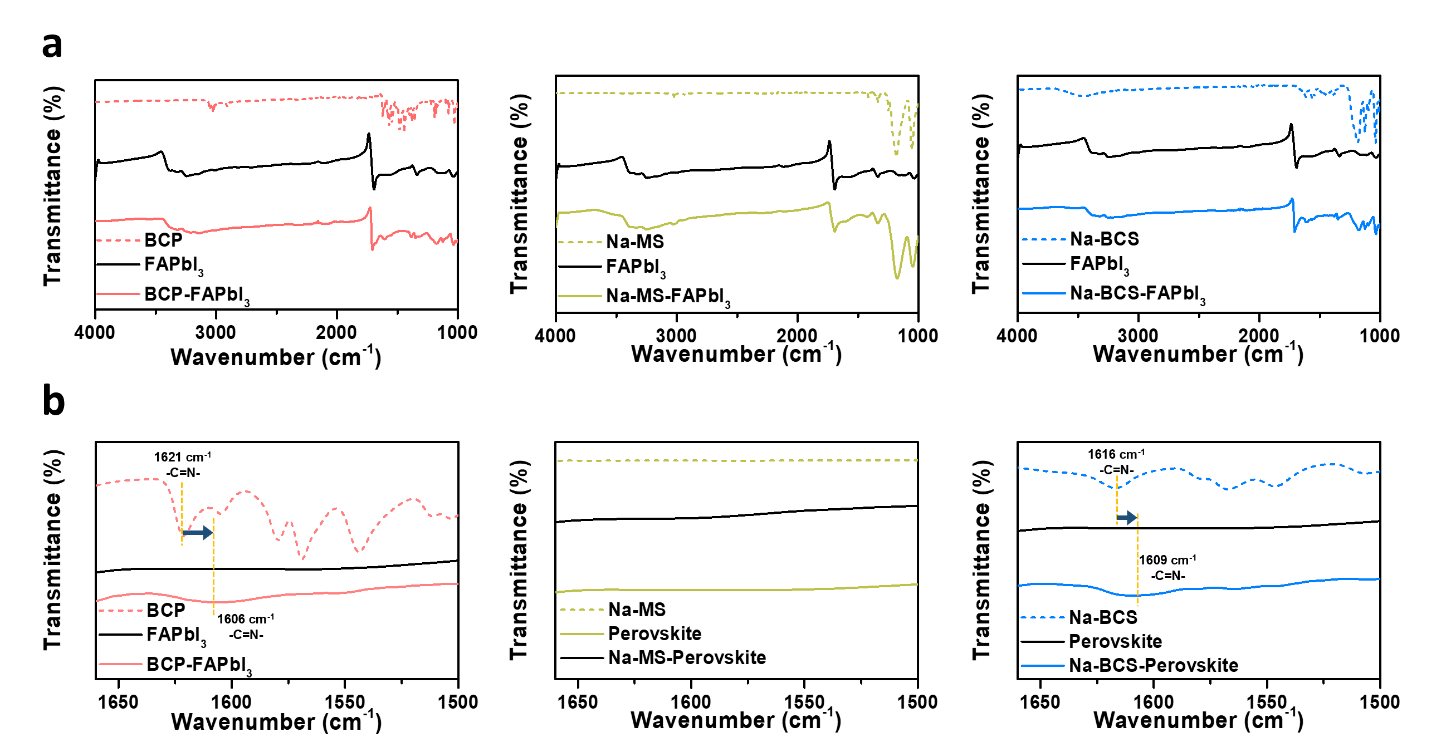
**Figure S6**. FT-IR spectra of BCP, Na-MS, and Na-BCS, together with FAPbI₃ and their corresponding mixtures with FAPbI₃.


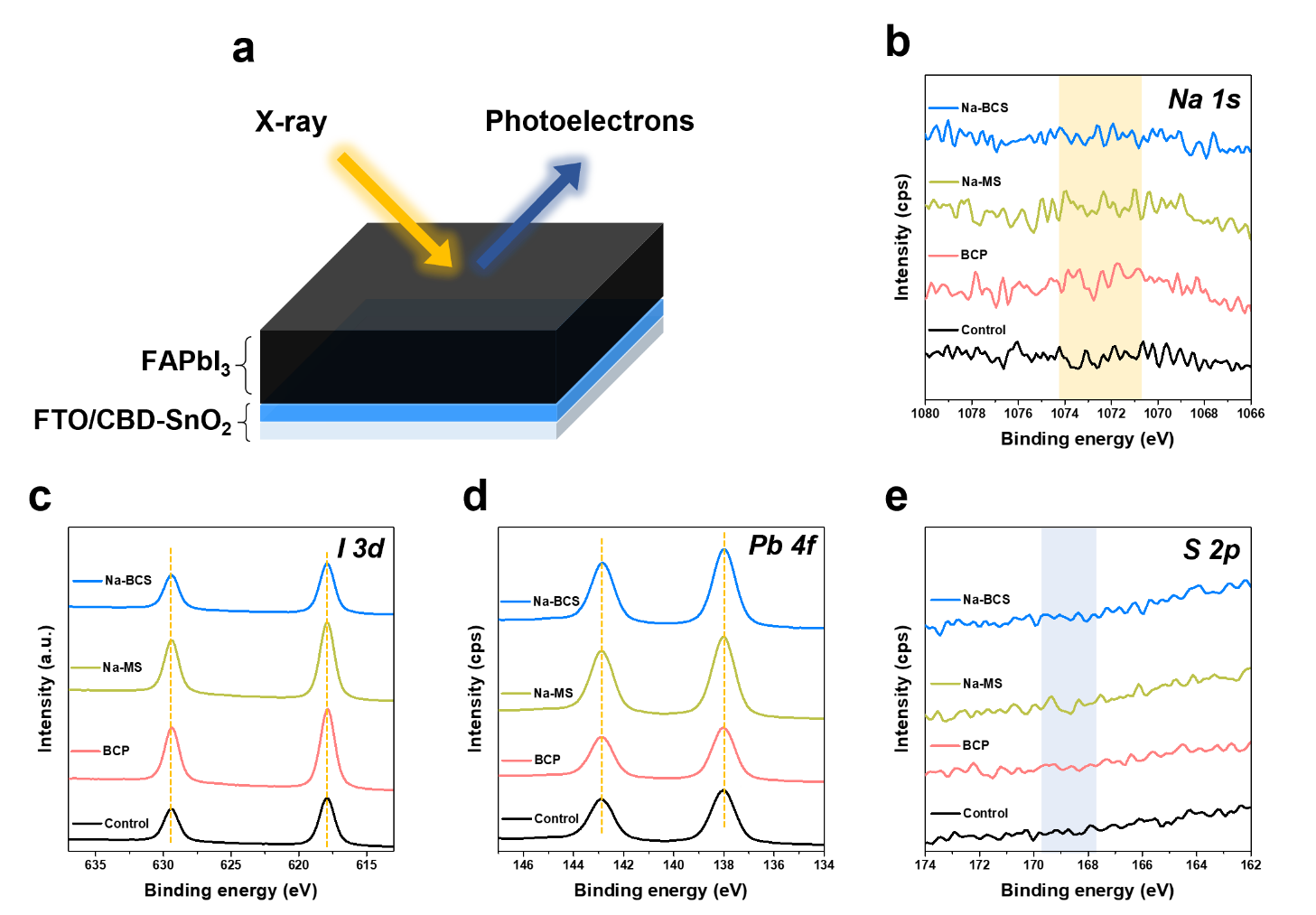
**Figure S7**. Top-surface XPS analysis of FAPbI₃ films deposited on FTO/CBD-SnO₂ substrates with buried-interface incorporation of BCP, Na-MS, or Na-BCS, together with the untreated control. (a) Schematic illustration of the top-surface XPS measurement geometry. (b) Na 1s, (c) Pb 4f, (d) I 3d spectra, (e) S 2p spectra.

**Figure S8**. UV-vis absorption spectra and (f) Urbach energy from UV-vis spectra for control, BCP, Na-MS, and Na-BCS samples.

**Figure S9**. Logarithm of absorption coefficient versus energy of the (a) control, and (b) BCP-, (c) Na-MS-, and (d) Na-BCS incorporated at buried interface.

**Figure S10**. Tauc plots of the control and buried interface modified perovskite films incorporating BCP, Na-MS, and Na-BCS.

**Figure S11.** Cross-sectional SEM images of the control and of samples incorporating BCP, Na-MS, or Na-BCS at the CBD-SnO₂/perovskite interface. Scale bars, 500 nm.

**Figure S12**. TRPL transient of perovskite films with varied thickness for control and samples (BCP-, Na-MS, and Na-BCS-incorporated at buried interface). Fabrication of all samples using a double-sided heterostructure architecture.


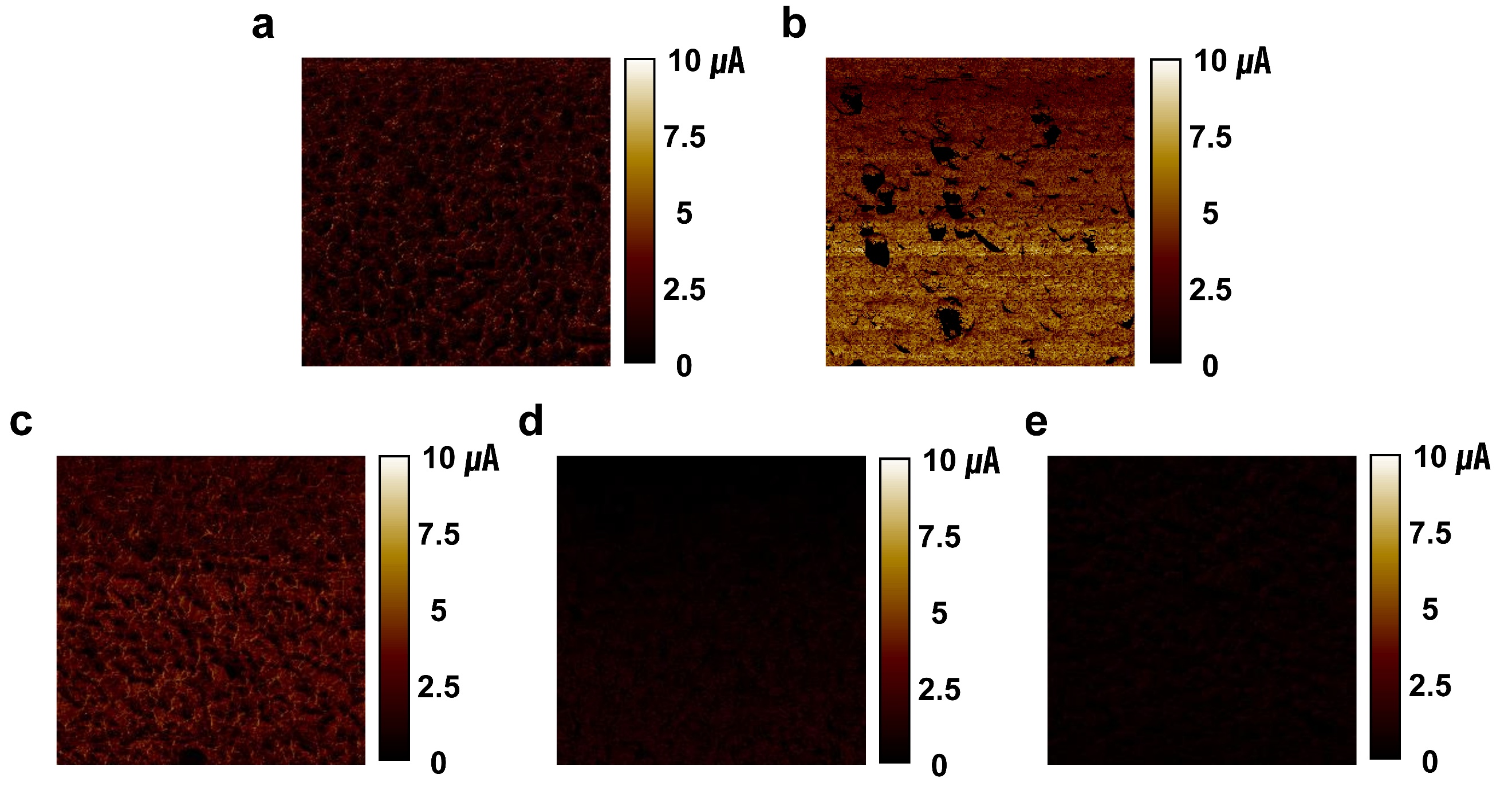


**Figure S13**. C-AFM characterizations of the SnO_2_ films as a function of Na-BCS concentration; (a) control, (b) 1mM Na-BCS, (c) 5mM Na-BCS, (d) 9mM Na-BCS, and 20mM Na-BCS.

**Figure S14.** UPS spectra around the secondary electron cut-offs of the control (CBD-SnO_2_), BCP, Na-MS and Na-BCS films.
**Figure S15**. Current density-QFLS plots of different conditions under various light intensities.

****Figure S16**. *J*_sc_, *V*_oc_, FF and PCE statistics of the control, BCP-, Na-MS-, and Na-BCS-treated devices.


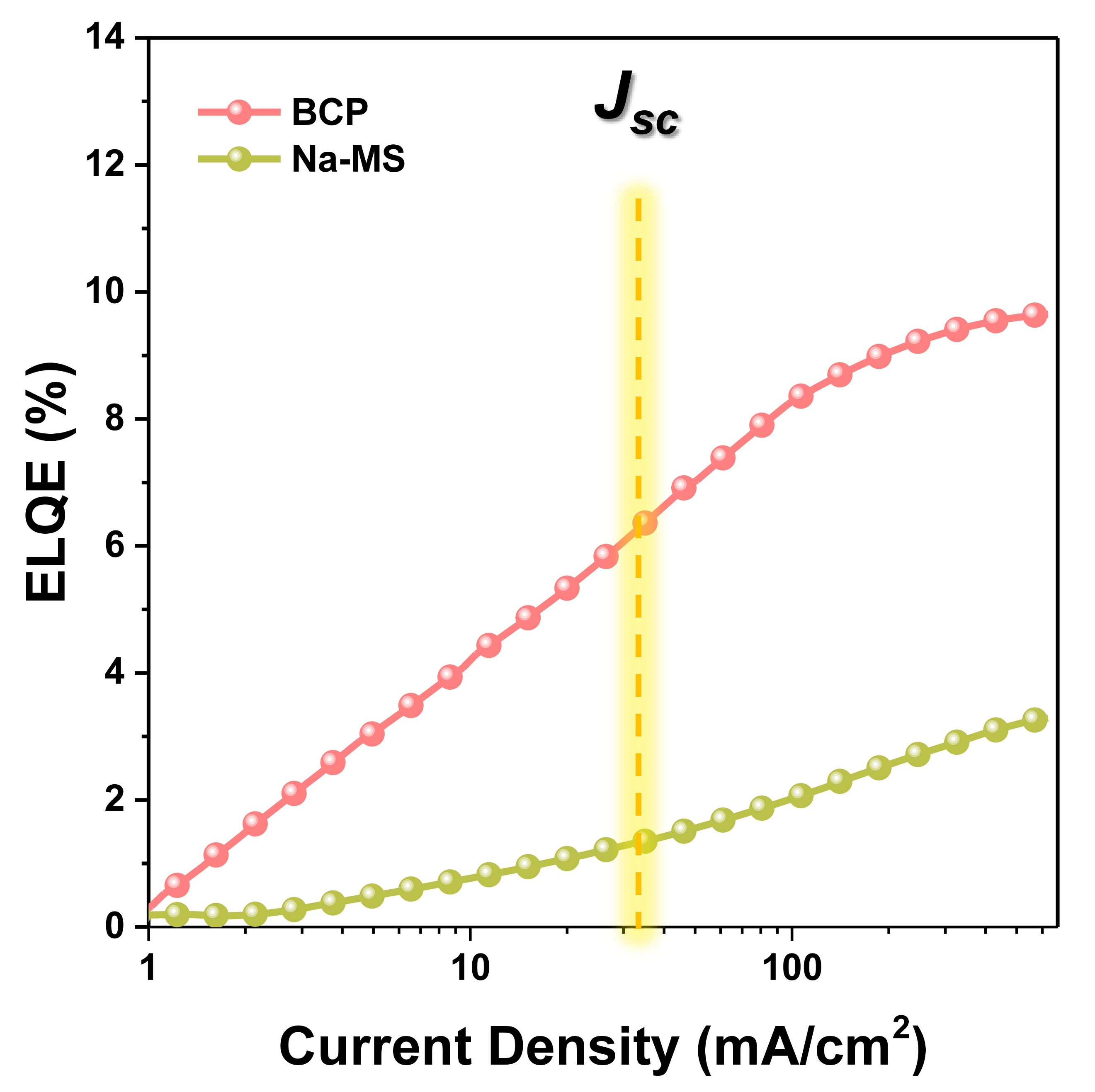


**Figure S17**. ELQE of the BCP and the Na-MS incorporated devices according to the injected current density.

**Figure S18**. Open-circuit voltage as a function of light intensity for the control, BCP, Na-MS, and Na-BCS devices.


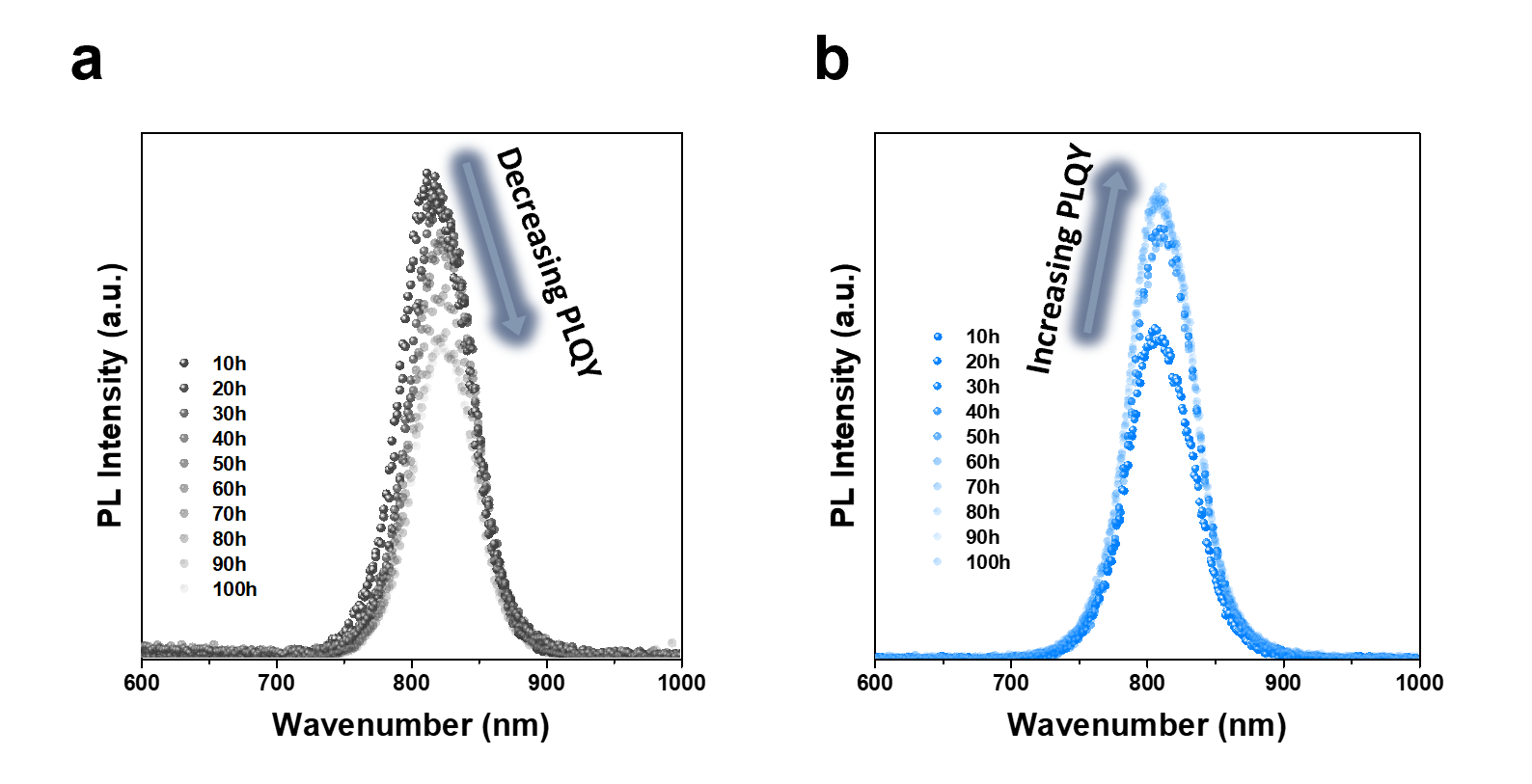


**Figure S19**. Time-dependent PL of the control and Na-BCS modified devices under continuous illumination conditions.

**Figure S20**. Comparison of TOF-SIMS profiles of Na-BCS-treated devices before and after 275 h of 1-sun LED illumination.

**Table S1**. TRPL fitting results for perovskite films for each condition. The carrier lifetime and average decay were calculated from TRPL measurements using the following equation:

F(t)=A_1_exp((-t)/τ_1_)+A_2_exp((-t)/τ_2_)+B
τ_avg_ = (A_1_τ_1_^2^ + Aτ_2_^2^)/(A_1_τ_1_ + A_2_τ_2_)

| **Devices** | **A_1_(%)** | **τ_1_ (ns)** | **A_2_(%)** | **τ_2_ (ns)** | **τ_avg_ (ns)** |
| --- | --- | --- | --- | --- | --- |
| Control | 40.2 | 108.7 | 59.8 | 696.1 | 640.6 |
| BCP | 38.6 | 13.4 | 61.4 | 1201.5 | 1131.5 |
| Na-MS | 33.9 | 185.9 | 66.1 | 1107.7 | 1034.4 |
| Na-BCS | 25.5 | 280.2 | 74.5 | 1890.2 | 1812.5 |

**Table S2**. Photovoltaic parameters for the devices depending on Na-BCS concentration.

| **Devices** | **Scan**  **direction** | **J_SC_**  **(mA cm^-2^)** | **V_OC_**  **(V)** | **FF**  **(%)** | **PCE**  **(%)** | **R-F average PCE**  **(%)** |
| --- | --- | --- | --- | --- | --- | --- |
| Control | fwd. | 25.93 | 1.073 | 80.62 | 22.43 | 22.44 |
|  | rev. | 26.18 | 1.056 | 81.20 | 22.45 |  |
| Na-BCS 1mM | fwd. | 26.15 | 1.161 | 81.62 | 24.77 | 24.85 |
|  | rev. | 26.18 | 1.159 | 82.20 | 24.94 |  |
| Na-BCS 5mM | fwd. | 26.19 | 1.185 | 83.83 | 26.02 | 25.94 |
|  | rev. | 26.21 | 1.186 | 83.25 | 25.87 |  |
| Na-BCS 9mM | fwd. | 25.95 | 1.175 | 83.17 | 25.35 | 25.48 |
|  | rev. | 26.08 | 1.177 | 83.45 | 25.61 |  |
| Na-BCS 13mM | fwd. | 25.97 | 1.174 | 81.23 | 24.80 | 24.85 |
|  | rev. | 26.03 | 1.175 | 81.45 | 24.90 |  |
| Na-BCS  20mM | fwd. | 25.97 | 1.172 | 78.19 | 23.79 | 23.51 |
|  | rev. | 26.03 | 1.168 | 76.45 | 23.24 |  |

**Table S3**. Photovoltaic parameters for the devices depending on BCP concentration.

| **Devices** | **Scan**  **direction** | **J_SC_**  **(mA cm^-2^)** | **V_OC_**  **(V)** | **FF**  **(%)** | **PCE**  **(%)** | **R-F average PCE**  **(%)** |
| --- | --- | --- | --- | --- | --- | --- |
| Control | fwd. | 25.93 | 1.073 | 80.62 | 22.43 | 22.44 |
|  | rev. | 26.18 | 1.056 | 81.20 | 22.45 |  |
| BCP  0.3mM | fwd. | 26.12 | 1.156 | 81.62 | 24.64 | 24.58 |
|  | rev. | 26.14 | 1.156 | 82.20 | 24.53 |  |
| BCP  0.6mM | fwd. | 26.11 | 1.172 | 81.83 | 25.04 | 25.07 |
|  | rev. | 26.14 | 1.168 | 82.25 | 25.11 |  |
| BCP  0.9mM | fwd. | 26.05 | 1.176 | 82.17 | 25.17 | 25.16 |
|  | rev. | 26.02 | 1.173 | 82.45 | 25.16 |  |
| BCP  1.2mM | fwd. | 25.95 | 1.171 | 80.19 | 24.36 | 24.37 |
|  | rev. | 25.92 | 1.170 | 80.45 | 24.39 |  |

**Table S4**. Photovoltaic parameters for the devices depending on Na-MS concentration.

| **Devices** | **Scan**  **direction** | **J_SC_**  **(mA cm^-2^)** | **V_OC_**  **(V)** | **FF**  **(%)** | **PCE**  **(%)** | **R-F average PCE**  **(%)** |
| --- | --- | --- | --- | --- | --- | --- |
| Control | fwd. | 25.93 | 1.073 | 80.62 | 22.43 | 22.44 |
|  | rev. | 26.18 | 1.056 | 81.20 | 22.45 |  |
| Na-MS  10mM | fwd. | 26.05 | 1.121 | 81.62 | 23.76 | 24.07 |
|  | rev. | 26.08 | 1.129 | 82.20 | 23.39 |  |
| Na-MS  20mM | fwd. | 26.11 | 1.128 | 83.43 | 24.57 | 24.62 |
|  | rev. | 26.12 | 1.131 | 83.52 | 24.67 |  |
| Na-MS  30mM | fwd. | 26.10 | 1.151 | 83.55 | 25.07 | 25.07 |
|  | rev. | 26.12 | 1.150 | 83.43 | 25.06 |  |
| Na-MS  40mM | fwd. | 25.97 | 1.148 | 82.19 | 24.50 | 24.44 |
|  | rev. | 26.03 | 1.150 | 81.45 | 24.38 |  |
